# Decadal Changes in Population Structures of Rare Oak Species *Quercus chungii*

**DOI:** 10.1002/ece3.70479

**Published:** 2024-10-19

**Authors:** Xueer Zhong, Wenbin Li, Zhenji Li, Yonghui Huang, Xinfeng Chen, Lihan Huang, Ya Wang, Yuxin Chen

**Affiliations:** ^1^ Key Laboratory of the Ministry of Education for Coastal and Wetland Ecosystems College of the Environment & Ecology, Xiamen University Xiamen China; ^2^ Administrative Office of Fujian Xiongjiang Huangchulin National Nature Reserve Minqing China

**Keywords:** endemic species, Fagaceae, life table, population dynamics, secondary forest, temporal change

## Abstract

*Quercus chungii*, a rare and endangered endemic tree species, is found exclusively in subtropical regions of China. Understanding the population structure and temporal dynamics of *Q. chungii* is pivotal for effective conservation and restoration of its populations and associated ecosystems. However, large knowledge gaps remain about its population structure and temporal change and its key demographic rates across size classes. In this study, we investigated the population structures of *Q. chungii* in 2013 and 2023 in a nature reserve specifically established to better conserve this species and its associated ecosystems. We found that *Q. chungii* increased in its overall abundance and tree size in the past decade, suggesting active regeneration and a rapid growth rate for this species and the effectiveness of past conservation efforts. The age structure in 2023 showed a pyramid shape, with a sharp decline in the numbers of individuals from germinated seeds to seedlings and from seedlings to saplings. These led to the low numbers of seedlings and saplings and high age‐specific death probabilities at the early developmental stages. These results indicated potential risks of future population decline. These risks may have already manifested over the past decade, as a high mortality rate during the seedling‐to‐sapling transition could be one of the primary reasons contributing to the decreased proportion of saplings in 2023 compared to 2013. We propose that future studies may benefit from in‐depth studies on the regeneration processes of *Q. chungii* by considering seed predation and germination under changing climate. This study improves the prediction of population development of *Q. chungii*, thereby offering theoretical guidance essential for its conservation.

## Graphical abstract

The endangered Chinese endemic tree species *Quercus chungii* showed increased abundance and size over the past decade in a nature reserve, indicating active regeneration and effectiveness of conservation efforts. However, its pyramid‐shaped age structure with low numbers of seedlings and saplings suggests potential risks of future population decline, emphasizing the need for in‐depth studies on regeneration processes.

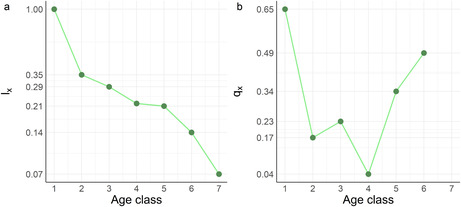

## 1 Introduction

Population structure and its dynamics are fundamental to understand and predict population development and critical for the conservation of rare and endangered species (Ezard et al. 2010; Harcombe 1987; Salguero‐Gomez and Gamelon 2021). *Quercus chungii* is a tree species of Fagaceae, endemic to China (Figure S1), and rare and endangered in Fujian Province (Jiang, Xu, and Deng 2019; Sun et al. 2021; Wang et al. 2011). The distribution area and population size of this species drastically reduced in the last century due to extensive logging for its high‐quality timber (Wang et al. 2011). Previous work has focused on the community structures, population spatial genetic pattern and distribution, and seed production and germination of *Q. chungii* (Chen 2004; Huang et al. 2010; Huang et al. 2017; Jiang, Xu, and Deng 2019; Li, Chen, and Liu 2010; Sun et al. 2021; Wang et al. 2011). However, large knowledge gaps remain about its age or size structures, temporal change, and the key demographic rates across age classes. Understanding the population structure and its temporal dynamics of *Q. chungii* is crucial for the conservation and restoration of its population, as well as the associated communities and ecosystems.

Age structure and life table are important tools to study population demographics (Ezard et al. 2010; Harcombe 1987; Jones 2021). Reproductive strategy and population development can be inferred from age structure by presenting the number of individuals in different developmental stages of a population. The information of age structure can be further used to construct a life table by calculating the key population parameters, such as age‐specific survival and mortality probabilities. Thus, life table is fundamental in exploring the heterogeneity of demographic processes across population developmental stages and predicting population dynamics and structures (Harcombe 1987; Jones 2021). A static or period life table is often used for long‐lived species, as it is difficult to track individuals from birth to death for such species (Jones 2021). It focuses on the fate of a population with mixed‐age classes during a particular time period. We can derive survivorship and mortality curves (Harcombe 1987) from life tables to assess mortality risks across age classes. There are usually three types of survivorship curves. Type I, characterized by a convex curve, signifies an increasing risk of death with age. Type II is a declining line, indicating a constant risk of death over all ages. Type III is a concave curve, indicating a decreasing risk of death with age. Survivorship curves of long‐lived, slow‐growing tree populations may not exactly fall into the three above types but show a mix of them (Harcombe 1987).

Seeds are essential for the survival and development of populations, as they determine the reproduction of seed plants and represent the beginning of any seed plant's life cycle (Willson and Traveset 2000). Although seed data are crucial for population analyses of plant species (Adams, Marsh, and Knox 2005; Salguero‐Gomez and Gamelon 2021), they are rarely incorporated into life table analyses of tree populations. Since seed data are usually difficult to obtain, most previous studies on tree population life tables set seedling or sapling as the starting stage (Farahat 2020; Ta et al. 2021; Wei et al. 2020; Wu et al. 2021; Zhang et al. 2023). Ignoring the seed stage may lead to inaccurate assessments and predictions of population demographics.

This study focuses on the population structure and its temporal change of *Q. chungii* in the Fujian Minqing Huangchulin National Nature Reserve, which was established in 1985 to better conserve this rare oak species and its associated communities and ecosystems (Li, Chen, and Liu 2010). We aim to study the following questions: (1) What is the current population structure of this species? (2) How did the population structures change from 2013 to 2023? (3) How did the mortality risk change across different size classes? We surveyed the population of *Q. chungii* in both 2013 and 2023 and compared the temporal changes in their population structures. We estimated the number of germinated seeds from a previous study of *Q. chungii* seed rain. To assess the mortality risks across size classes, we derived the survivorship and mortality curves from the static life table. This study will improve the understanding of the current status of the endemic and endangered *Q. chungii* tree species and the prediction of its future development, thus providing important guidance for its conservation.

## 2 Materials and Methods

### 2.1 Study Site

We surveyed the population structures and dynamics of *Q. chungii* in Minqing Huangchulin National Nature Reserve located in Fujian Province, China (26°5′29″ N ~ 26°22′41″ N, 118°39′38″ E ~ 118°51′19″ E, with an elevation range of 100–595 m) (Figure S1). This area has the most intense distribution of *Q. chungii* in the world (Li, Chen, and Liu 2010). The reserve was specifically established to conserve *Q. chungii* and its associated communities and ecosystems (Li, Chen, and Liu 2010) by prohibiting logging, minimizing human disturbance, and monitoring both its populations and associated ecosystems. The reserve has a mid‐subtropical maritime monsoon climate, with an average annual temperature of 17.5°C, an average annual sunshine duration of 1871.4 h, a frost‐free period of 294 days, and an average annual precipitation of 1400–1900 mm. The forest soil is predominantly mountainous red soil (Li, Chen, and Liu 2010).

### 2.2 Population Survey

We established two permanent plots in the nature reserve to survey the population of *Q. chungii* in 2013. One plot is 40 m × 40 m located near Tangxia village, and the other is 20 m × 30 m located at Fengshiling. Both plots are located within areas with the most concentrated population or where the largest stems of *Q. chungii* were found, in addition to having a known logging history. We measured every tree with a DBH (diameter at breast height) ≥ 2.5 cm within the plots in 2013.

To assess the temporal changes in population structures, we re‐surveyed the population in the two permanent plots in 2023. To obtain a more complete structure of population, we measured all individuals, including seedlings with a DBH < 2.5 cm, within the permanent plots. We established four additional 20 m × 20 m plots near Tangxia village in the nature reserve to measure all individuals of *Q. chungii*. Thus, in 2023, we surveyed the population structure of *Q. chungii* in a total area of 3800 $m^{2}$ with relatively dense distribution of the focal species.

### 2.3 Population Age Structure

We used the size structure of *Q. chungii* to represent its age structure, as it is difficult to directly measure age (e.g., by dendrochronological analysis) from such a rare and endangered tree species, and size structures are commonly used to infer population age structures of tree species (Harcombe 1987; Ta et al. 2021; Wei et al. 2020; Wu et al. 2021; Zhang et al. 2023). We separated the population into seven age classes based on the estimated relationship between tree age and size from logged *Q. chungii* in this nature reserve (Yang 2005) and previous studies on the growth (Liu 2005) and population structures (Wang et al. 2011) of this species. The seven age classes are (1) germinated seeds, (2) seedlings with DBH < 2.5 cm, (3) saplings with 2.5 cm ≤ DBH < 12.5 cm, (4) medium trees with 12.5 cm ≤ DBH < 22.5 cm, (5) medium trees with 22.5 cm ≤ DBH < 32.5 cm, (6) large trees with 32.5 cm ≤ DBH < 42.5 cm, and (7) large trees with DBH ≥ 42.5 cm. Germinated seeds were estimated from a previous study on seed rain and germination of *Q. chungii* (see below), while data of age classes 2–7 were from a field survey of this study. We obtained all the age classes in 2023, while only age classes 3–7 were available in 2013.

We estimated the number of germinated seeds by assuming that a tree population with a larger total basal area will produce more germinated seeds (Greene and Johnson 1994; Wright et al. 2021), and this proportional relationship remains constant through time:

(1) $$\frac{\text{Number of germinated seeds in}\;2007}{\text{Species basal area in}\;2007}=\frac{\text{Number of germinated seeds in}\;2023}{\text{Species basal area in}\;2023}$$

The number of germinated seeds in 2007 was from a previous study (Huang et al. 2010). This study set up 60 1 m^2^ seed traps in three 20 m × 20 m plots in the same nature reserve in 2007 and counted the number of germinated seeds. Species basal area in 2023 were from this study. If the total basal area of the community did not change from 2007 to 2023, we can estimate the number of germinated seeds in 2023 from the following equation:

(2) $$\frac{\text{Number of germinated seeds in}\;2007}{\text{Relative species basal area in}\;2007}=\frac{\text{Number of germinated seeds in}\;2023}{\text{Relative species basal area in}\;2023}$$

The relative species basal area of *Q. chungii* in 2007 was from a previous study on *Q. chungii* communities in the same nature reserve (Wang et al. 2011); the relative species basal area in 2023 was from this study. We also estimated the number of germinated seeds in 2023 by assuming a constant proportional relationship between the number of germinated seeds and the average of relative species basal area and abundance, or the number of germinated seeds did not change through time. These two alternative analyses yielded qualitatively similar results; hence, we did not present them in this paper.

We utilized bootstrap resampling with 10,000 repetitions to estimate the 95% confidence interval (95% CI) of population numbers for each age class.

### 2.4 Temporal Change in Population Structures

We selected the age classes 3–7 (DBH ≥ 2.5 cm) that had available data both in 2013 and 2023 to compare the temporal changes in population numbers in each age class. We also compared the overall difference in basal areas, abundances, and frequencies by aggregating data from these age classes. Frequency was calculated as the proportion of 10 m × 10 m plots with *Q. chungii* presence. To estimate the uncertainties of these differences, we resampled the 10 m × 10 m plots with replacement, calculated the difference in basal areas, abundances, and frequencies between years, and repeated this process 10,000 times. With this bootstrap resampling, we constructed the 95% CI of the differences in these statistics. If the 95% CI did not overlap with zero, we concluded that there was a significant difference in the corresponding statistics between years. We estimated the 95% CIs for the means of abundance, frequency, and basal area in each year using bootstrap resampling with 10,000 repetitions.

### 2.5 Construction and Analysis of Static Life Table

We constructed the static life table of the *Q. chungii* population by following the definition in Jones (2021) (Table 1). Survivorship and mortality curves were derived from the life table to assess mortality risks across age classes. We applied the smoothing approach (Wratten and Fry 1980) to deal with the issue of the negative number of individuals who died during the observational period (_*n*_ *D*_*x*_). For example, if the observed number of individuals alive in age class 3 (_*n*_ *N*_3_ = 21) is smaller than that in age class 4 (_*n*_ *N*_4_ = 34), then the estimated _*n*_ *D*_3_ = _*n*_ *N*_3_ −_*n*_ *N*_4_ would be a negative value. The smoothing approach assumes an equal proportional change in _*n*_ *N*_*x*_ in an interval centered at such age class (2–4 in this case). We calculated the average value (μ) and the difference between the maximum and minimum values (∆) of _*n*_ *N*_*x*_ from the three age classes. The estimated values of _*n*_ *N*_*x*_ after smoothing are _*n*_ *N*_2_*=μ−∆3, _*n*_ *N*_3_*=μ, and _*n*_ *N*_4_*=μ+∆3.

**TABLE 1:** Definition and calculation of coefficients in static life table.

Coefficient	Definition and calculation
*x*	Age at the start of each age class. We assume *x*s are 1–7 for age classes 1–7
*n*	Interval length between age classes, which is assumed to be one in this study
_*n*_ *N*_*x*_	Observed number of individuals alive in the middle of an age interval. _*n*_ *N*_*x*_ in the first age class was calculated by assuming a proportional relationship between the number of germinated seeds and species basal area (see Section 2); _*n*_ *N*_*x*_ of the other age classes (2–7) are from field survey
_*n*_ *N*_x_*	Estimate of _*n*_ *N*_*x*_ by smoothing (see Section 2)
_*n*_ *D*_*x*_	Number of individuals died during the observational period: _*n*_ *D*_*x*_ = _*n*_ *N*_x_*−_*n*_ *N*_x+n_*. All individuals are assumed to be dead in the final age class
_*n*_ *m*_*x*_	Age‐specific mortality rate: _*n*_ *m*_*x*_ = _*n*_ *D*_*x*_/_*n*_ *N*_*x*_
_*n*_ *a*_*x*_	Average length of survival time in each age range, which is assumed to be 0.5
_*n*_ *q*_*x*_	Probability of death from ages *x* to *x + n*: _*n*_ *q*_*x*_ = (*n·*_*n*_ *m*_*x*_)/(1 + (*n* −_*n*_ *a*_*x*_)·_*n*_ *m*_*x*_). _*n*_ *q*_*x*_ of the last age class is assumed to be one
_*n*_ *p*_*x*_	Probability of survival from ages *x* to *x + n*: _*n*_ *p*_*x*_ = 1−_*n*_ *q*_*x*_.
*l*_*x*_	The relative number of individuals entering the interval at age *x*. *l*_*x+n*_ *= l*_*x*_·_*n*_ *p*_*x*_. The initial population size entering the smallest age class (*l*_1_) is assumed to be one in this study. Thus, *l*_*x*_ can be interpreted as the probability of survival for age class *x*
_*n*_ *d*_*x*_	Relative number of individuals died from ages *x* to *x + n*: _*n*_ *d*_*x*_ *= l*_*x*_ − *l*_*x+*1_

All the above analyses were performed in R 4.3.1 (R Development Core Team 2023). The bootstrap method was implemented by using the “*boot*” package (Canty and Ripley 2022), and the graphs were created using the “ggplot2” package (Wickham 2016).

## 3 Results

### 3.1 Population Structure and Its Temporal Change

In 2013, we recorded 28 individuals of *Q. chungii* with DBH ≥ 2.5 cm within 2200 m^2^. In 2023, we found 144 individuals of *Q. chungii* with DBH ≥ 1 cm and 37 individuals with DBH <1 cm within 3800 m^2^. Only one of the seedlings with DBH < 1 cm was produced from seed, while the others were sprouted from the roots of established trees.

**FIGURE 1:**
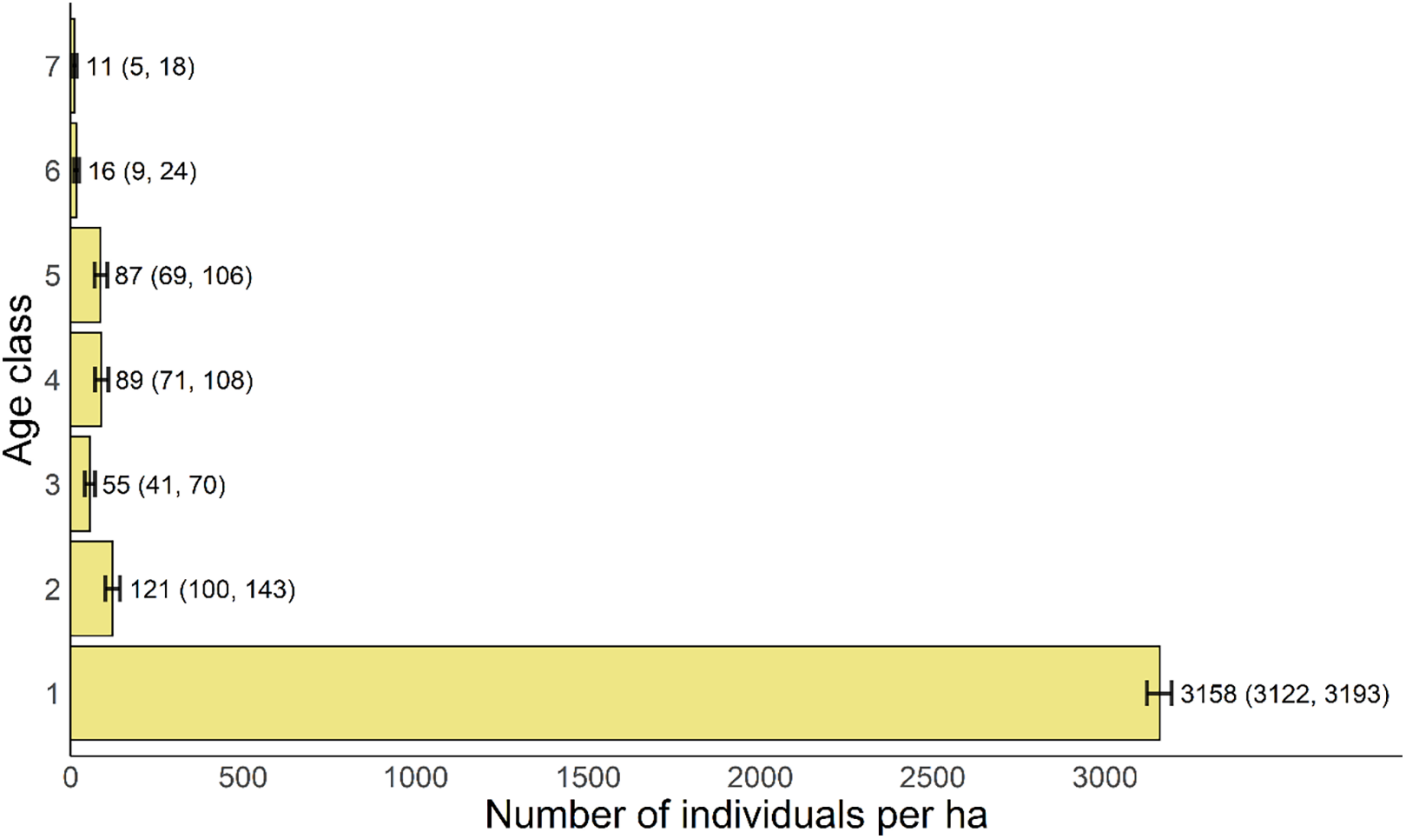
Age structure of *Quercus chungii* population in 2023. The numbers adjacent to the bars represent the count of individuals per hectare within each age class. The population number in the first age class was calculated by assuming a proportional relationship between the number of germinated seeds and overall basal area of the population; the population numbers of other age classes (2–7) are from field surveys. Detailed information about the age structures is shown in Table 2. We estimated the 95% CI of population numbers in each age class by bootstrap resampling with 10,000 repetitions. Numbers within the brackets represent the lower and upper limits of 95% CI, respectively. The bars represent the mean values derived from resampling.

The population structure in 2023 showed a pyramidal structure (Figure 1). The focal species could produce a large number of germinated seeds (age class 1), but the number of seedlings (age class 2) dramatically decreased, suggesting high risks of death from stages of seed to seedling. The pattern is similar from stages of seedling to sapling (age class 3). The numbers of medium trees at age classes 4–5 were larger than those of saplings and large trees (age classes 6–7).

**FIGURE 2:**
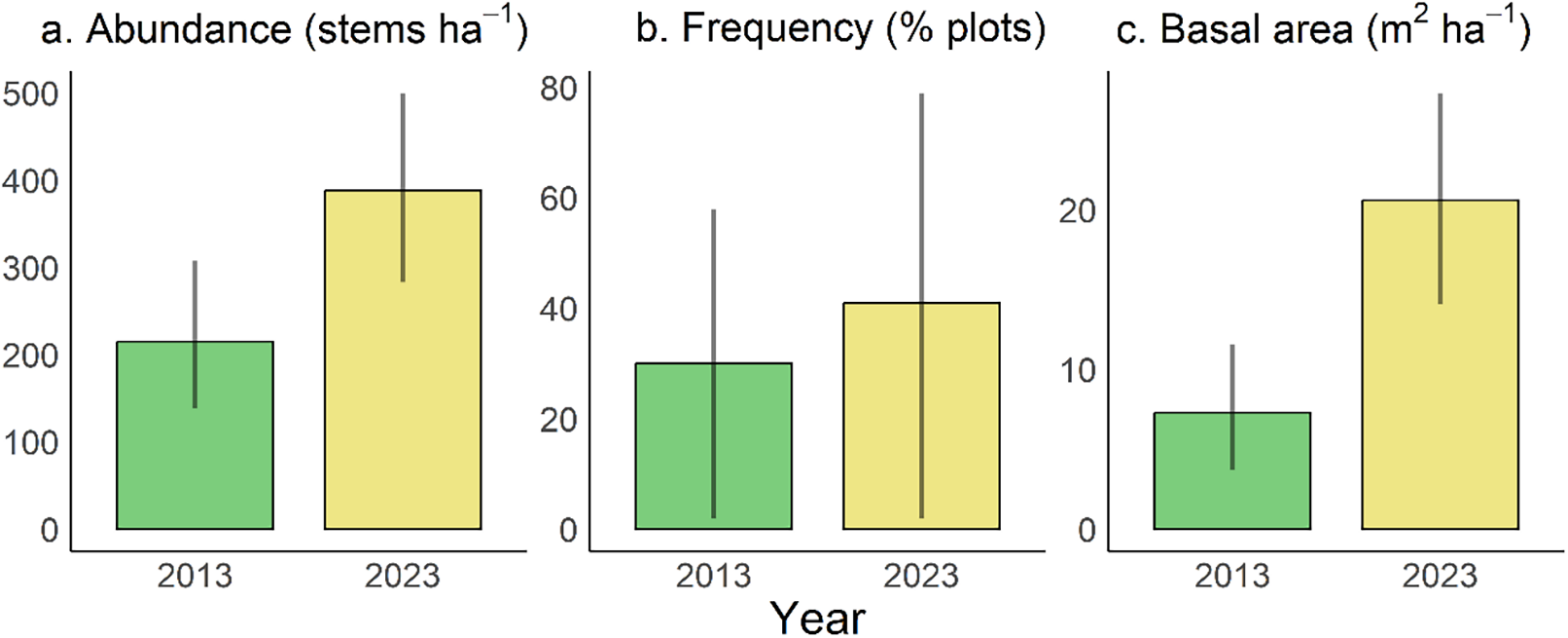
*Quercus chungii* increased in abundance (a), frequency (b), and basal area (c) from 2013 to 2023. Frequency was calculated as the percentage of 10 m × 10 m plots in which *Quercus chungii* was observed. We used bootstrap resampling with 10,000 repetitions to estimate the uncertainties of the mean abundance, frequency, and basal area in each year, as well as their differences between years. We determined that there is a significant difference between years if the 95% CI of the difference does not overlap with zero. The bars and their error bars represent the mean values and 95% CI, respectively, derived from the resampling.

**FIGURE 3:**
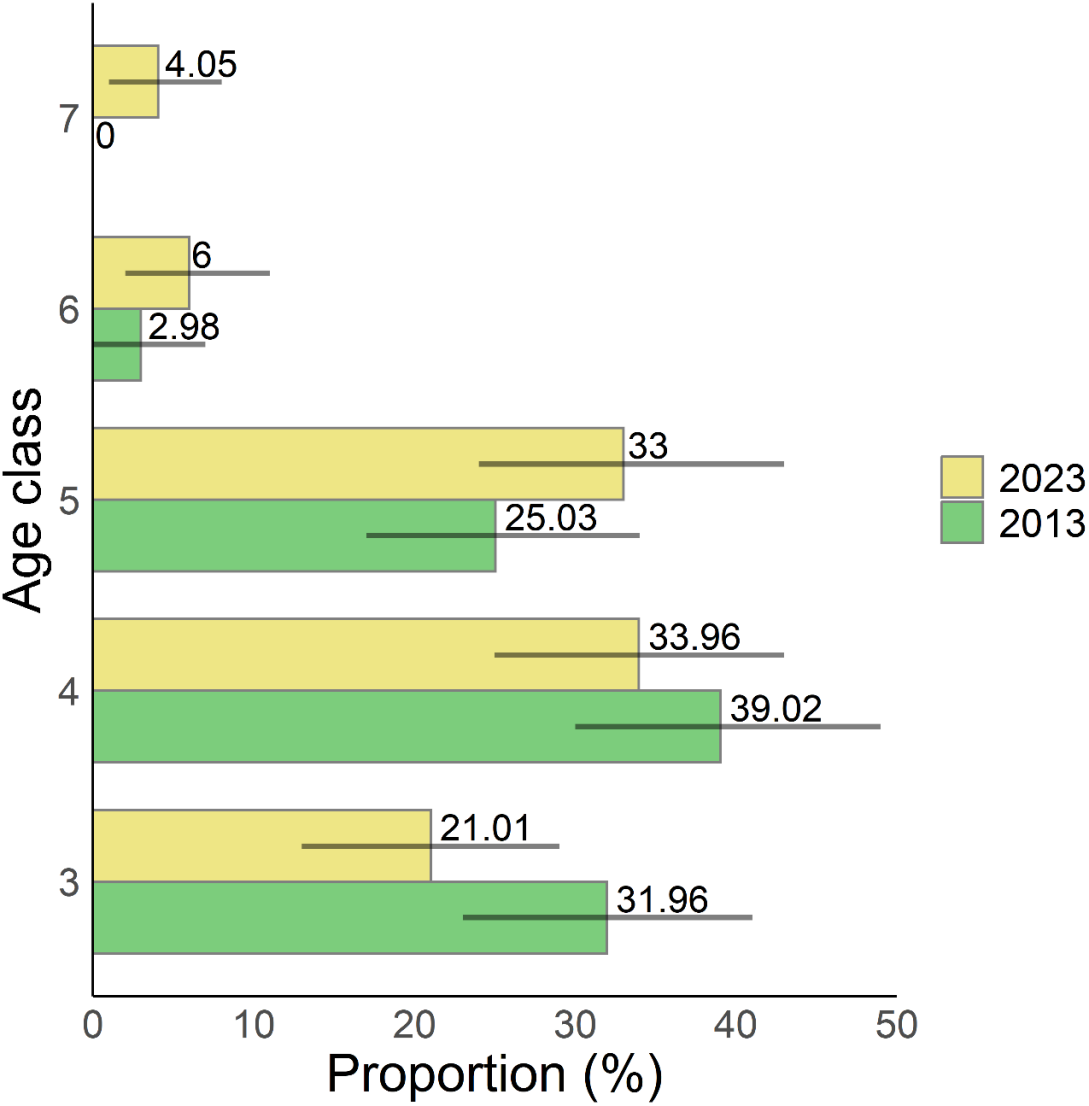
Difference in age structures of the *Quercus chungii* population between 2013 and 2023. Proportions of individuals were calculated for age classes 3–7 surveyed in both years. Numbers besides bars represent the proportions within each age class in each year. Detailed information about the age structures is shown in Table 2. Error bars indicate the 95% CI of population numbers in each age class, derived from bootstrap resampling with 10,000 repetitions. The bars represent the mean values derived from resampling.

During the past decade, from 2013 to 2023, the increase in the abundance of *Q. chungii* was marginally significant (Figure 2a) and its frequency slightly increased (Figure 2b). The basal area increased significantly during this period (Figure 2c). For individuals with DBH≥ 2.5 cm (age classes 3–7) surveyed in both years, the proportion of saplings tended to decrease from 2013 to 2023, whereas the proportions of trees with larger sizes (age classes 5–7) tended to increase over time (Figure 3), although not statistically significant. These results suggest evident tree growth over the past decade, which is consistent with the findings of an increase in basal area (Figure 2c).

### 3.2 Life Table and Survivorship Curve

Based on the static life table in 2023 (Table 2), we found that the number of individuals who died from germinated seeds to seedlings (_*n*_ *D*_1_) was the highest, resulting in the correspondingly highest age‐specific mortality rate (_*n*_ *m*_1_) and age‐specific death probability (_*n*_ *q*_1_). The smallest coefficients associated with these mortality risks occurred in small‐medium trees at age class 4.

**TABLE 2:** Static life table of *Quercus chungii* population in 2023.

Age classes (cm)	*x*	_*n*_ *N*_*x*_	_*n*_ *N*_x_*	_*n*_ *D*_*x*_	_*n*_ *a*_*x*_	_*n*_ *m*_*x*_	_*n*_ *q*_*x*_	_*n*_ *p*_*x*_	*l*_*x*_	_*n*_ *d*_*x*_
Germinated seeds	1	5506	5506	5464	0.500	0.992	0.663	0.337	1.000	0.663
DBH < 2.5	2	46	42	8	0.500	0.190	0.174	0.826	0.337	0.059
2.5 ≤ DBH < 12.5	3	21	34	9	0.500	0.265	0.234	0.766	0.278	0.065
12.5 ≤ DBH < 22.5	4	34	25	1	0.500	0.040	0.039	0.961	0.213	0.008
22.5 ≤ DBH < 32.5	5	33	24	10	0.500	0.417	0.345	0.655	0.205	0.071
32.5 ≤ DBH < 42.5	6	6	14	9	0.500	0.643	0.487	0.513	0.134	0.065
DBH ≥ 42.5	7	4	5	5	0.000	1.000	1.000	0.000	0.069	0.069

*Note:* The calculation of static life table is based on population survey within a total area of 3800 m^2^.

**FIGURE 4:**
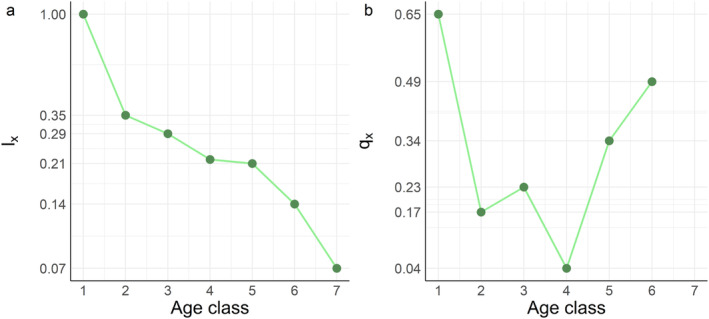
Survivorship (a) and mortality (b) curves of *Quercus chungii* population in 2023. The probability of survival to age class *x* (*l*_*x*_) was plotted on a log‐transformed scale but shown with its original values, as proportional changes in *l*_*x*_ can be better compared with the changes in age‐specific probabilities of death (*q*_*x*_). Detailed information about the age structures is shown in Table 2.

The survivorship curve showed a reverse sigmoid pattern (Figure 4a). That is, the decreasing rate of age‐specific survival probability (*l*_*x*_) slowed down in smaller age classes (1–4; i.e., shown as Type III concave curve), but increased in speed for larger age classes (4–6; i.e., shown as Type I convex curve). This pattern of survivorship trend was the result of a U‐shape mortality curve (Figure 4b). That is, the age‐specific death probability (*q*_*x*_) first decreased then increased with age classes. The transition of the temporal direction of death probability occurred in small‐medium trees at age class 4.

## 4 Discussion

The *Q. chungii* forest is one of the typical representatives of the evergreen broad‐leaved forest in the southern margin of the mid‐subtropical zone of China and also an important habitat for terrestrial wild animals. However, it has undergone decline in the past due to human disturbances (Li et al. 2010; Wang et al. 2011). In this study, we found that *Q. chungii* has increased in its overall abundance and tree size over the past decade, suggesting active regeneration and a rapid growth rate for *Q. chungii*. These results indicate the effectiveness of conservation efforts in establishing the nature reserve. However, the in‐depth analyses of population structures warn potential risks of the future population development of *Q. chungii*.

We found the age structure of the *Q. chungii* population in 2023 showed a pyramidal shape, with a sharp decline in the numbers of individuals from germinated seeds to seedlings and from seedlings to saplings. Saplings, a key stage for determining the future population, had a low number of individuals. These led to the high age‐specific death probabilities at the early developmental stages. These results indicate potential risks of population decline in the future. The observed decline in sapling proportion from 2013 to 2023 may signify a population decline trend within the species. This decrease could be attributed to either accelerated ontogenetic progression of saplings into larger size classes or elevated mortality rates during early developmental stages and within the sapling cohort. If mortality factors predominate, it underscores a potential population decline over the past decade.

The seed‐to‐seedling stage may be the most important period limiting the regeneration of the *Q. chungii* population, as the highest mortality probabilities occurred during this transition from germinated seeds to seedlings. This is also consistent with our observation in the field. We found a very low density of seedlings with DBH < 1 cm in 2023. Only one of them may be produced from seed, while the others were evidently sprouted from the roots of established trees. The scarcity of seedlings produced from seeds was already observed more than a decade ago (Wang et al. 2011), which may be one of the reasons driving the decadal decline in the proportion of saplings observed in this study. The dependence on clonal reproduction can be one of the most important factors driving the highly aggregated spatial distribution of *Q. chungii* in natural forests (Wang et al. 2011). The aggregated distribution may increase the risk from pests and diseases. The reduced genetic diversity due to clonal reproduction may further increase these risks (Bagchi et al. 2014; Gilbert and Webb 2007; Liang et al. 2016; Liu et al. 2015), thus threatening the health and development of the *Q. chungii* population in the future.

Seeds are crucial to the survival and distribution of seed plant populations. Consequently, a more precise analysis of the population structure of forest tree species necessitates the inclusion of all age classes, encompassing the seed stage (Doak, Thomson, and Jules 2022). When the seed stage is rarely incorporated into life table analyses of tree populations, we filled this gap by incorporating the seedling stage into life table analyses of a rare oak species' populations and its population structure analysis. We estimated the number of germinated seeds from historical data on seeds. We assumed that trees with a larger basal area will produce more germinated seeds, because larger trees can obtain more resources and produce more seeds (S and Johnson 1994; Wright et al. 2021). We acknowledge that the production and germination of seeds are complex processes determined by multiple factors, such as climate, soil, animal predation, plant physiology, and human disturbance (Baskin and Baskin 2014; Donohue et al. 2010). The accuracy of our estimation approach thus relies on how these biotic and abiotic factors change in the past decade within the nature reserve. We thought that these changes should be slow due to the relative short temporal duration and the prevention of human activities by the administrative office of the nature reserve. The findings of a high number of germinated seeds were also confirmed from the recent field observation by the officers of the nature reserve. But most of the seeds were found to be eaten by boars and rodents.

Despite the high predation risks from mammals, the structural and physiological traits of *Q. chungii* may also drive the severe decline in population numbers from seeds to seedlings. The germination pore of *Q. chungii* seed locates at a key place for water transportation, which may make it vulnerable to rapid water deficits (Sun et al. 2021). Moreover, *Q. chungii* has a substantially delayed shoot emergence following a fast root emergence. The breakage of this shoot dormancy often needs 3–5 months with warm temperature (Sun et al. 2021). These seed traits can make *Q. chungii* very sensitive to precipitation and temperature abnormality, thus reducing the probability of transition from seeds to seedlings.

The age‐specific death probability of *Q. chungii* first decreased then increased with age classes, resulting in a U‐shape pattern. Large and tall trees are usually more vulnerable to disease, wind, and other causes of death (Fernández‐de‐Uña et al. 2023; Lu et al. 2021), which may drive the increasing mortality probability from medium‐sized to large trees.

## 5 Conclusions and Prospects

In this study, we aimed to investigate the decadal changes in population structures of the rare and endangered oak species *Q. chungii*. Our findings reveal significant increases in tree density and size, indicating a positive trend in population development over the studied period. A closer examination of the transition probabilities from seeds to seedlings and saplings uncovers the potential risk of regeneration failure and future population decline. The low success rate from seeds to seedlings underscores the fragility of *Q. chungii*'s population. We advocate for long‐term population monitoring to gain insights into *Q. chungii*'s growth patterns and potential threats, including the intricate impacts of multitrophic interactions and climate change. Furthermore, targeted fencing around selected maternal trees for protection, along with monitoring and collecting seeds for artificial propagation, can supplement the natural seedling establishment process.

Our study, therefore, not only improves the prediction of *Q. chungii*'s population development but also provides valuable theoretical guidance for the development of targeted conservation strategies aimed at mitigating the risks identified and ensuring the long‐term persistence of this endangered species. However, our research is confined to a limited scope and a few selected populations located in relatively small areas. To fully comprehend the species' population development dynamics and its implications for the ecosystem as a whole, future endeavors must embark on a broader scale by encompassing a more diverse array of populations across various habitats and ecological contexts.

## Author Contributions

**Xueer Zhong:** conceptualization (equal), data curation (lead), formal analysis (lead), investigation (equal), methodology (equal), visualization (lead), writing – original draft (lead), writing – review and editing (equal). **Wenbin Li:** conceptualization (lead), investigation (equal), methodology (equal), project administration (equal), supervision (lead), writing – original draft (lead), writing – review and editing (lead). **Zhenji Li:** conceptualization (equal), funding acquisition (lead), investigation (equal), methodology (supporting), project administration (equal), writing – review and editing (equal). **Yonghui Huang:** investigation (supporting), methodology (equal), project administration (supporting), writing – review and editing (equal). **Xinfeng Chen:** investigation (equal), project administration (supporting), writing – review and editing (supporting). **Lihan Huang:** investigation (equal), project administration (supporting), writing – review and editing (supporting). **Ya Wang:** investigation (equal), writing – review and editing (supporting). **Yuxin Chen:** conceptualization (equal), funding acquisition (lead), investigation (equal), writing – original draft (supporting), writing – review and editing (supporting).

## Conflicts of Interest

The authors declare no conflicts of interest.

## Supporting information


Data S1.

## Data Availability

The datasets that supporting the findings of this study are available in the figshare at https://figshare.com/s/d9adcf4b14c940891840.

## References

[ece370479-bib-0001] Adams, V. M. , D. M. Marsh , and J. S. Knox . 2005. “Importance of the Seed Bank for Population Viability and Population Monitoring in a Threatened Wetland Herb.” Biological Conservation 124: 425–436.

[ece370479-bib-0002] Bagchi, R. , R. E. Gallery , S. Gripenberg , et al. 2014. “Pathogens and Insect Herbivores Drive Rainforest Plant Diversity and Composition.” Nature 506, no. 7486: 85–88. 24463522 10.1038/nature12911

[ece370479-bib-0003] Baskin, C. C. , and J. M. Baskin . 2014. Seeds: Ecology, Biogeography, and, Evolution of Dormancy and Germination. San Diego, CA: Academic Press.

[ece370479-bib-0004] Canty, A. , and B. Ripley . 2022. “Boot: Bootstrap R (S‐Plus) Functions.” R Package Version 1.3‐28.1.

[ece370479-bib-0005] Chen, S. 2004. “Changes in Species Diversity of Plants in *Cyclobalanopsis chungii* Forest During the Course of Restoration.” Journal of Zhejiang Forestry College 21, no. 3: 258–262.

[ece370479-bib-0500] Doak, D. F., D. , D. Thomson , and E. S. Jules . 2002. “Population Viability Analysis for Plants: Understanding the Demographic Consequences of Seed Banks for Population Health.” In Population viability analysis, edited by S. R. Beissinger and D. R. McCullough , 151–162. Chicago: The University of Chicago Press.

[ece370479-bib-0007] Donohue, K. , R. Rubio de Casas , L. Burghardt , K. Kovach , and C. G. Willis . 2010. “Germination, Postgermination Adaptation, and Species Ecological Ranges.” Annual Review of Ecology, Evolution, and Systematics 41: 293–319.

[ece370479-bib-0008] Ezard, T. H. G. , J. M. Bullock , H. J. Dalgleish , et al. 2010. “Matrix Models for a Changeable World: The Importance of Transient Dynamics in Population Management.” Journal of Applied Ecology 47, no. 3: 515–523.

[ece370479-bib-0009] Farahat, E. A. 2020. “Age Structure and Static Life Tables of the Endangered *Juniperus phoenicea* L. in North Sinai Mountains, Egypt: Implication for Conservation.” Journal of Mountain Science 17: 2170–2178.

[ece370479-bib-0010] Fernández‐de‐Uña, L. , J. Martínez‐Vilalta , R. Poyatos , M. Mencuccini , and N. G. McDowell . 2023. “The Role of Height‐Driven Constraints and Compensations on Tree Vulnerability to Drought.” New Phytologist 239, no. 6: 2083–2098. 37485545 10.1111/nph.19130

[ece370479-bib-0011] Gilbert, G. S. , and C. O. Webb . 2007. “Phylogenetic Signal in Plant Pathogen‐Host Range.” Proceedings of the National Academy of Sciences of the United States of America 104, no. 12: 4979–4983. 17360396 10.1073/pnas.0607968104 PMC1829250

[ece370479-bib-0012] Greene, D. , and E. A. Johnson . 1994. “Estimating the Mean Annual Seed Production of Trees.” Ecology 75, no. 3: 642–647.

[ece370479-bib-0013] Harcombe, P. A. 1987. “Tree Life Tables.” Bioscience 37, no. 8: 557–568.

[ece370479-bib-0014] Huang, Y. , X. Ma , K. Zhuang , M. Liu , and D. Huang . 2010. “Seed Rain and Soil Seed Bank of *Cyclobalanopsis chungii* Forest in Minqing, Fujian Province.” Journal of Tropical and Subtropical Botany 18, no. 1: 68–74.

[ece370479-bib-0015] Huang, Y. , K. Zhuang , P. Wu , X. Ma , X. Lai , and W. Tang . 2017. “Seed Germination and Growth Characteristics of *Cyclobalanopsis chungii* .” Chinese Journal of Ecology 36, no. 5: 1251–1258.

[ece370479-bib-0016] Jiang, X. , G. Xu , and M. Deng . 2019. “Spatial Genetic Patterns and Distribution Dynamics of the Rare Oak *Quercus chungii*: Implications for Biodiversity Conservation in Southeast China.” Forests 10, no. 9: 821.

[ece370479-bib-0017] Jones, O. R. 2021. “Life Tables: Construction and Interpretation.” In Demographic Methods Across the Tree of Life, edited by R. Salguero‐Gómez and M. Gamelon , 151–162. Oxford: Oxford University Press.

[ece370479-bib-0018] Li, Z. , X. Chen , and C. Liu . 2010. Comprehensive Scientific Investigation Report of Fujian Xiongjiang Huangchulin Nature Reserve. Xiamen: Xiamen University Press.

[ece370479-bib-0019] Liang, M. , X. Liu , G. S. Gilbert , et al. 2016. “Adult Trees Cause Density‐Dependent Mortality in Conspecific Seedlings by Regulating the Frequency of Pathogenic Soil Fungi.” Ecology Letters 19, no. 12: 1448–1456. 27790825 10.1111/ele.12694

[ece370479-bib-0020] Liu, C. 2005. “Community Characteristics and Growth of Natural Forest and Plantation of *Cyclobalanopsis chungii* .” Journal of Zhejiang A &F University 22, no. 1: 56–60.

[ece370479-bib-0021] Liu, X. , R. S. Etienne , M. Liang , Y. Wang , and S. Yu . 2015. “Experimental Evidence for an Intraspecific Janzen‐Connell Effect Mediated by Soil Biota.” Ecology 96, no. 3: 662–671. 26236863 10.1890/14-0014.1

[ece370479-bib-0022] Lu, R. , Y. Qiao , J. Wang , et al. 2021. “The U‐Shaped Pattern of Size‐Dependent Mortality and Its Correlated Factors in a Subtropical Monsoon Evergreen Forest.” Journal of Ecology 109: 2421–2433.

[ece370479-bib-0023] R Development Core Team . 2023. R: A Language and Environment for Statistical Computing. Vienna: R Foundation for Statistical Computing. http://www.r‐project.org.

[ece370479-bib-0024] Salguero‐Gomez, R. , and M. Gamelon . 2021. Demographic Methods Across the Tree of Life. Oxford: Oxford University Press.

[ece370479-bib-0025] Sun, X. , Y. Song , B. Ge , X. Dai , and G. Kozlowski . 2021. “Intermediate Epicotyl Physiological Dormancy in the Recalcitrant Seed of *Quercus chungii* F.P.Metcalf With the Elongated Cotyledonary Petiole.” Forests 12, no. 3: 263.

[ece370479-bib-0026] Ta, F. , X. Liu , D. Huang , et al. 2021. “Quantitative Dynamics of *Picea crassifolia* Population in Dayekou Basin of Qilian Mountains.” Acta Ecologica Sinica 41, no. 17: 6871–6882.

[ece370479-bib-0027] Wang, Y. , P. Wu , W. Rong , M. Xiang , and H. Xian . 2011. “Community Characteristics of Cyclobalanopsis Chungii Forest in Mingqing Nature Reserve.” Journal of Fujian Agricultural and Forestry University 40, no. 1: 37–42.

[ece370479-bib-0028] Wei, X. , Y. Ye , X. Lin , Y. Cui , F. Zeng , and F. Wang . 2020. “Population Status and Conservation of an Extremely Small Population Species *Euryodendron excelsum* .” Chinese Journal of Plant Ecology 44, no. 12: 1236–1246.

[ece370479-bib-0029] Wickham, H. 2016. ggplot2: Elegant Graphics for Data Analysis. New York: Springer‐Verlag.

[ece370479-bib-0030] Willson, M. F. , and A. Traveset . 2000. “The Ecology of Seed Dispersal.” In Seeds: The Ecology of Regeneration in Plant Communities, edited by M. Fenner , 85–110. Wallingford: CABI.

[ece370479-bib-0031] Wratten, S. D. , and G. L. A. Fry . 1980. Field and Laboratory Exercises in Ecology. London: Edward Aroold.

[ece370479-bib-0032] Wright, M. C. , P. J. Mantgem , N. L. Stephenson , A. J. Das , and J. E. Keeley . 2021. “Seed Production Patterns of Surviving Sierra Nevada Conifers Show Minimal Change Following Drought.” Forest Ecology and Management 480: 118598.

[ece370479-bib-0033] Wu, Q. , F. Zang , C. Li , et al. 2021. “Population Structure and Dynamics of Endangered *Populus wulianensis* .” Acta Ecologica Sinica 41, no. 12: 5016–5025.

[ece370479-bib-0034] Yang, C. 2005. “A Study on the Growth of Natural *Cyclobalanopsis chungii* Forest in Fujian Province.” East China Forest Management 19, no. 3: 29–33.

[ece370479-bib-0035] Zhang, X. , Y. Xie , X. Wu , Y. Li , and S. Xiao . 2023. “Population Structure and Dynamic Characteristics of Wild Plant Species With Extremely Small Populations of *Camptotheca acuminata* in Mingxi, Fujian Province, China.” Ecology and Environmental Sciences 32, no. 6: 1037–1044.

