# Supplementary material for: Decadal Changes in Population Structures of Rare Oak Species Quercus chungii
Source: Ecol Evol. 2024 Oct 19;14(10):e70479. doi: 10.1002/ece3.70479 (PMC11489618; doi:10.1002/ece3.70479)
Supplement: Supplementary file 1 — Data S1. [file ECE3-14-e70479-s001.docx]

**Supplementary Material**

**
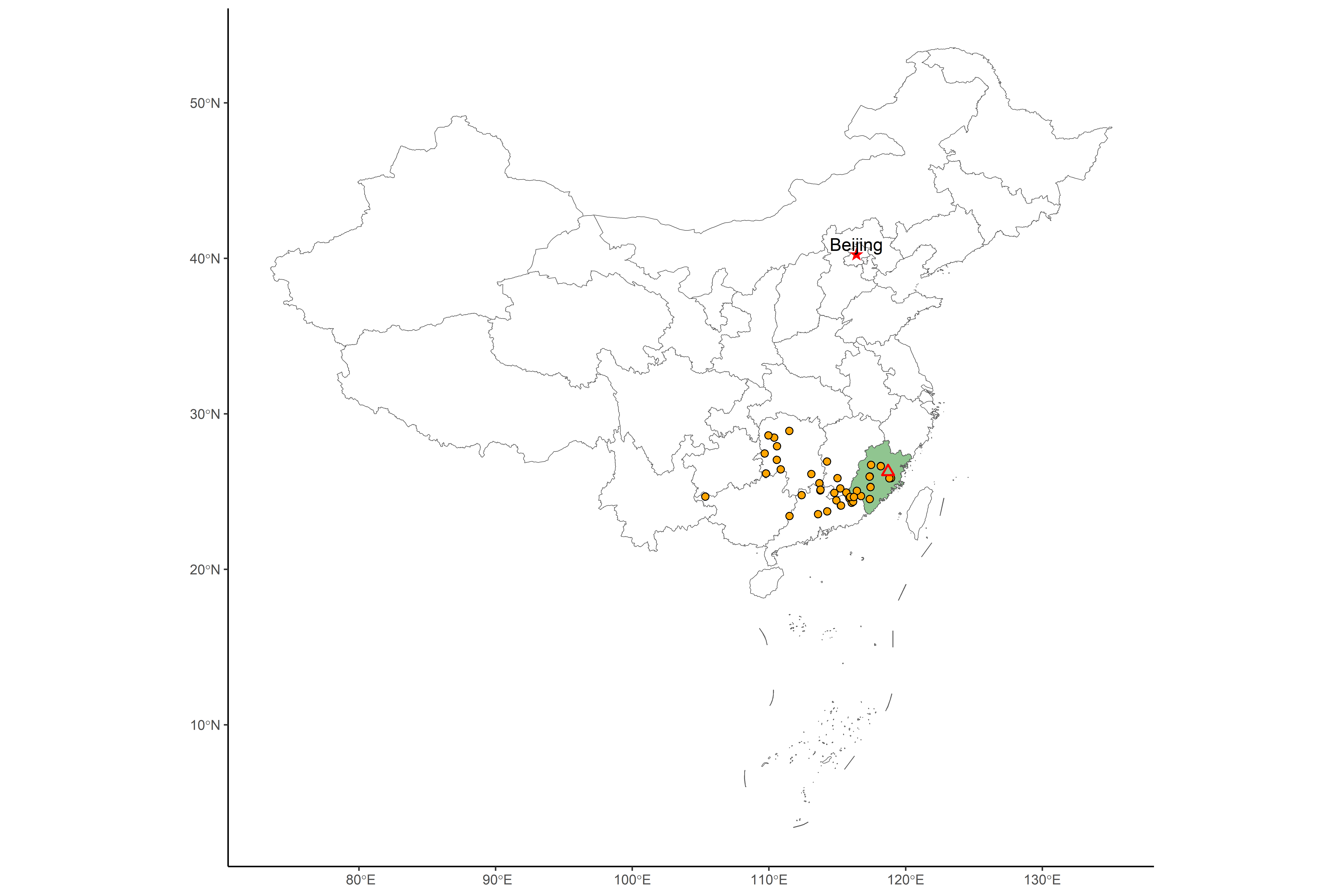
**

**Figure S1.** The location of Minqing Huangchulin National Nature Reserve (red triangle) and the distribution of *Quercus chungii* (orange points) in China. The green area represents Fujian Province. Data of species occurrence are from the Global Biodiversity Information Facility (GBIF, DOI: https://doi.org/10.15468/dl.qg7k49; data accessed in 18 September 2024).
